# 2204 Characterizing specialized pro-resolving lipid mediators and synthesis pathways in veterans with peripheral artery disease

**DOI:** 10.1017/cts.2018.53

**Published:** 2018-11-21

**Authors:** Joel Ramirez, Greg J. Zahner, Sukaynah A. Khetani, Kimberly A. Spaulding, Nancy K. Hills, S. Marlene Grenon

**Affiliations:** University California, San Francisco, CA, USA

## Abstract

OBJECTIVES/SPECIFIC AIMS: Specialized pro-resolving lipid mediators (SPM) actively counter proinflammatory cascades. A deficit of SPMs is one possible mechanism through which inflammation leads to the development of atherosclerotic disease. The purpose of this study is to characterize the profiles of intermediates of SPM synthesis pathways and end-product SPMs in the plasma of patients with peripheral artery disease (PAD). METHODS/STUDY POPULATION: A cross-sectional sample of 52 patients with PAD was recruited at the San Francisco Veterans Affairs Medical Center. PAD was defined as the presence of claudication symptoms and an ankle-brachial index <0.9, or a history of revascularization for claudication. Patients were excluded if they were taking immunosuppressive medications, had a severe acute illness (infection, surgery, illness, critical limb ischemia) within the last 30 days, or had severe hepatic, renal, or nonvascular inflammatory disease. Intermediates of SPM synthesis pathways and end-product SPMs were measured in plasma samples of patients by liquid chromatography-tandem mass spectrometry. RESULTS/ANTICIPATED RESULTS: The average age of the cohort was 69±6.3 and patient comorbidities reflected common comorbidities associated with PAD (hypertension 96%, hyperlipidemia 87%, diabetes mellitus 42%, coronary artery disease 34%). Rutherford categories, measurements of PAD symptom severity, ranged from 0 to III (0 10%, I 40%, II 27%, III 23%). Three EPA products were measured: 18-hydroxyeicosapentaenoic acid (18-HEPE), resolvin E1 (RvE1), and resolvin E2 (RvE2). 18-HEPE, an intermediate of SPM synthesis, was detectable in the plasma of every patient (median: 105 pg/mL, IQR: 54.9–195), whereas the SPM end-products, RvE1 and RvE2, were only detectable in 6 and 10 patients, respectively. In total, 7 DHA products were measured: 14-hydroxydocosahexaenoic acid (14-HDHA), 17-HDHA, resolvin D1 (RvD1), resolvin D2 (RvD2), protectin D1, protectin DX, and maresin 1. The intermediates 14-HDHA (median: 6546 pg/mL, IQR: 3329–12061) and 17-HDHA (median: 644 pg/mL, IQR: 340–1056) were detectable in the plasma of every patient. However, the end-products RvD1, RvD2, protectin D1, protecin DX, and maresin 1 were identified in less than half of the cohort. DISCUSSION/SIGNIFICANCE OF IMPACT: We report the presence of several intermediates of SPM synthesis pathways (18-HEPE, 14-HDHA, and 17-HDHA) in every patient but the presence of SPM end-products in only a limited portion of the cohort. These results suggest that some patients with PAD may have a deficit of SPMs. Further investigation is required to better understand the role of SPMs and mediators of resolution of inflammation in PAD.

